# Systematic reviews of patient-reported outcome measures (PROMs): table templates for effective communication

**DOI:** 10.1007/s11136-025-04058-y

**Published:** 2025-09-03

**Authors:** Ellen B. M. Elsman, Maarten Boers, Caroline B. Terwee, Dorcas Beaton, Inger Abma, Olalekan Lee Aiyegbusi, Alessandro Chiarotto, Kirstie Haywood, Karen Matvienko-Sikar, Ava Mehdipour, Diana M. Oosterveer, Lidwine B. Mokkink, Martin Offringa

**Affiliations:** 1 Department of Epidemiology and Data Science, Amsterdam UMC, Vrije Universiteit Amsterdam, De Boelelaan 1117, 1081 HV Amsterdam, The Netherlands; 2 Amsterdam Public Health Research Institute, Methodology, Amsterdam, The Netherlands; 3 Child Health Evaluative Sciences, The Hospital for Sick Children Research Institute, Toronto, ON Canada; 4 Institute of Work and Health, Toronto, ON Canada; 5 IQ Health Science Department, Radboud University Medical Center, Nijmegen, The Netherlands; 6 Centre for Patient Reported Outcomes Research (CPROR), University of Birmingham, Birmingham, UK; 7 Department of General Practice, Erasmus MC, University Medical Center, Rotterdam, The Netherlands; 8 Warwick Research in Nursing, Warwick Medical School, University of Warwick, Coventry, UK; 9 School of Public Health, University College Cork, Cork, Ireland; 10 School of Nursing, Trinity Western University, Langley, BC Canada; 11 Basalt, Department of Rehabilitation, Leiden, The Netherlands; 12 Institute of Health Policy, Management and Evaluation, University of Toronto, Toronto, ON Canada

**Keywords:** Systematic reviews, Data visualization, Patient-reported outcome measures (PROMs), COSMIN, OMERACT, Measurement properties

## Abstract

**Purpose:**

Systematic reviews of outcome measurement instruments (OMIs) are an important tool to guide the selection of OMIs for research and clinical practice. However, presenting the large amount of complex data pertaining both to the quality of each study (i.e., risk of bias) as well as the quality of the instrument (i.e., measurement properties), along with the underpinning certainty of evidence, is challenging. Here, we aim to provide guidance on optimizing data presentation in OMI systematic reviews, specifically focusing on patient-reported outcome measures (PROMs).

**Methods:**

A multidisciplinary team of experts in OMI systematic reviews, research reporting, and data visualization built on existing table templates from OMERACT and the COSMIN initiative, to align with reporting items in a recently developed reporting guideline for systematic reviews of OMIs: PRISMA-COSMIN for OMIs 2024. To enhance clarity and usability, we applied data visualization principles by reducing non-essential elements and improving interpretability through structured layouts and concise explanatory text.

**Results:**

We present eight templates for reporting PROM systematic review results: three pertain to PROM characteristics, two to studies’ characteristics, two to the evaluation of measurement properties, and one to the summary of findings. We also provide recommendations on whether to include these templates in the review’s main manuscript or in the supplementary materials. Word versions of these templates can be downloaded from www.prisma-cosmin.ca and www.cosmin.nl.

**Conclusion:**

Templates complementing the PRISMA-COSMIN for OMIs 2024 reporting guidance can be used to standardize and enhance the clarity and usefulness of OMI systematic reviews focusing on PROMs. They comprise a comprehensive set of tools to effectively report OMI systematic reviews, in service of end-users who are selecting OMIs.

**Supplementary Information:**

The online version contains supplementary material available at 10.1007/s11136-025-04058-y.

## Introduction

Systematic reviews of outcome measurement instruments (OMIs) are an important tool in the evidence-based selection of an OMI for use in research and clinical practice. Such reviews synthesize data from primary studies on OMIs’ measurement properties, feasibility, and/or interpretability, providing insight into the suitability of an OMI for a particular purpose [1]. The number of OMI systematic reviews has increased steadily over the past two decades, with 113 such systematic reviews published in 2024 [2]. To help researchers conduct OMI systematic reviews, several organizations have developed guidelines and methodology, including Outcome Measures in Rheumatology (OMERACT) [3] and the COnsensus-based Standards for the selection of health Measurement INstruments (COSMIN) initiative [1, 4]. COSMIN guidelines in particular, have been widely accessed and adopted by researchers (as indicated by over 2500 citations since its publication in 2018), and both OMERACT and COSMIN guidelines have recently been updated [4, 5].

However, conducting OMI systematic reviews is challenging and time consuming, because multiple measurement properties per OMI need to be considered. In fact, reviewers need to conduct multiple reviews in parallel (i.e., one for each measurement property). The results of each of these reviews need to be synthesized to eventually formulate a conclusion on the quality of one or multiple OMIs, including the certainty of the evidence. Moreover, before these steps, the methodological quality of each study needs to be assessed through a risk of bias assessment [1, 3–5]. A large amount of data thus needs to be processed and reported in an efficient and effective manner.

To ensure proper conduct of systematic reviews, the COSMIN guideline for conducting systematic reviews of PROM was published along with an extensive user manual, which guides a reviewer through the process of conducting a systematic review [6]. To ensure transparency of this process, COSMIN has developed the “COSMIN review management file” to present the data processed in systematic reviews of patient-reported outcome measures (PROMs) [7]. This file can be included in the supplementary materials of an article. Similarly, OMERACT has data extraction and summary tables for reporting the evidence on measurement properties [8]. The final step of the review is to report the work. To guide the reporting of OMI systematic reviews, an extension of the PRISMA (Preferred Reporting Items for Systematic reviews and Meta-Analyses) guideline specifically for these types of reviews was recently published: *PRISMA-COSMIN for OMIs 2024* [9]. However, while drafting the Explanation & Elaboration (E&E) document for *PRISMA-COSMIN for OMIs 2024*, we observed large variability in how results were presented in tables, making it difficult to extract good examples from the published literature. Many tables did not adhere to two fundamental design principles: *clear vision* and *clear understanding* [10, 11].

Here we provide table templates designed to enhance the clarity of OMI systematic review results, specifically focusing on PROMs. These templates apply visual design principles to ensure effective data presentation [10–13] and align with both *PRISMA-COSMIN for OMIs 2024* [9] as well as the COSMIN review management file but can be used for non-COSMIN-based reviews as well. Clear vision, summarized as optimum data-to-supporting-structure ratio, can be achieved by reducing unnecessary visual elements (such as grids) and optimizing the layout of text and numbers for readability. Clear understanding, summarized as an information structure that effectively conveys key messages, is facilitated through logical data organization.

## Development of the templates

*PRISMA-COSMIN for OMIs 2024* [9] provided the framework to identify which table templates for PROM systematic review results needed to be developed. This reporting guideline includes eight results topics represented by one or multiple reporting items (Table 1). The PRISMA-COSMIN for OMIs flow diagram for study selection has previously been published [9], so table templates needed to be developed for the remaining topics.

**Table 1:** Overview of available and newly developed templates for relevant reporting items in *PRISMA-COSMIN for OMIs 2024* [9]

Section and topic	#	Checklist item*	Template (recommended location)†
*Results*			
Study selection	22a	Describe the results of the search and selection process, from the number of records identified in the search to the number of study reports included in the review, ideally using a flow diagram. If applicable, also report the final number of OMIs included and the number of study reports relevant to each OMI. [T]	See published PRISMA-COSMIN for OMIs flow diagram [9] (M)
	22b	Cite study reports that might appear to meet the inclusion criteria, but which were excluded, and explain why they were excluded.	N/A
OMI characteristics	23a	Present characteristics of each included OMI, with appropriate references. [T]	Template 1 (M)
	23b	If applicable, present interpretability aspects for each included OMI. [T]	Template 2 (S)
	23c	If applicable, present feasibility aspects for each included OMI. [T]	Template 3 (S)
Study characteristics	24	Cite each included study report evaluating one or more measurement properties and present its characteristics. [T]	Template 4 (M/S) and 5 (M/S)
Risk of bias in studies	25	Present assessments of risk of bias for each included study. [T]	Template 6 (S) and 7 (S)
Results of individual studies	26	For all measurement properties, present for each study: (a) the reported result; (b) the rating against quality criteria, ideally using structured tables or plots. [T]	Template 6 (S) and 7 (S)
Results of syntheses	27a	Present results of all syntheses conducted. For each measurement property of an OMI, present: (a) the summarized or pooled result; (b) the overall rating against quality criteria. [T]	Template 6 (S), 7 (S), and 8/8a (M)
	27b	If applicable, present results of all investigations of possible causes of inconsistency among study results.	N/A
	27c	If applicable, present results of all sensitivity analyses conducted to assess the robustness of the synthesized results.	N/A
Certainty of evidence	28	Present assessments of certainty (or confidence) in the body of evidence for each measurement property of an OMI assessed. [T]	Template 6 (S), 7 (S), and 8/8a (M)
Recommendations	29	If appropriate, make recommendations for suitable OMIs for a particular use.	N/A

N/A: not applicable

*If an item is marked with [T], a template for data visualization is available. These templates can be downloaded from www.prisma-cosmin.ca or www.cosmin.nl.

^†^M: main manuscript; S: supplementary files

To develop the table templates, we combined and refined existing templates from the COSMIN initiative and OMERACT [7, 14]. The “COSMIN review management file” is an Excel file containing multiple worksheets for data extraction, including PROM details, study results, risk of bias ratings, measurement properties ratings, and certainty of evidence gradings (available from www.cosmin.nl). It is primarily a tool to support data extraction in PROM systematic reviews and results need to be transferred into example tables provided in the COSMIN manual [6]. These example tables can still be improved for clarity and usability, as they do not adhere to the clear vision and clear understanding design principles. In addition, OMERACT has created a knowledge translation table to communicate systematic review results, called the SOMP (Summary Of Measurement Properties) [14]. Although the SOMP provides a high-level structured summary, similar to Template 6 and 7 of this paper, it does not include all data items that require reporting by *PRISMA-COSMIN for OMIs 2024* [9]. We note that OMERACT has developed other reporting tools [8] that were not used as sources for our current template development.

We applied key principles of data visualization and table design to ensure that the presentation of results facilitates interpretation by end-users [10–13]. To ensure that the developed templates were both methodologically sound and visually effective, we assembled a multidisciplinary team of researchers with expertise in systematic reviews of PROMs, research reporting, and data visualization. These experts were involved in the development of *PRISMA-COSMIN for OMIs 2024* to which these templates are central [9]. They first met at the PRISMA-COSMIN end-of-project meeting to design the templates and were subsequently involved during 15 months of refining and internal peer review of earlier template versions. Their individual contributions resulted in co-authorship on this paper. This collaborative and comprehensive approach warranted that the templates not only adhere to PROM systematic review methodology but also follow best practices for structuring complex data in a way that enhances clarity and usability.

To illustrate how these templates should be structured and formatted, we populated them with fictional sample data. The sample data only serves to demonstrate the layout and organization of the tables, without representing actual study findings. In general, the content aligns with the COSMIN guideline for systematic reviews of PROMs, specifying what data should be extracted and how it should be synthesized. Therefore, the templates presented here are for PROM-based reviews but can be modified for other types of OMIs.

In designing the layout of the templates, we used very light background shading to put extra emphasis on the rows and to prevent the use of rules (horizontal lines) between rows. In general, rules were used as little as possible and made light gray and thin to avoid obscuring the data. In all tables (except Template 9), the following cell margins were used: top 0.1 cm, bottom 0.05 cm, left 0.15 cm, and right 0.15 cm. This allows enough white space around text and numbers for easy reading, while also maximizing the space in a table. The larger project group ensured that the arrangement of data facilitated comparisons and is easy to read.

In terms of text formatting, we ensured that the precision matched the purpose of the data and prevented high precision whenever this was irrelevant. Therefore, it is often sufficient to present results with two significant numbers only: i.e., percentages as integer and one decimal point for data in the range of 1–10. Text is mostly left aligned, whereas numbers are right (or decimally) aligned. Moreover, we ensured that numbers were placed each element in their own cell wherever possible. In the examples, we used smart line breaks to split text at natural pauses, avoiding automatic word wrapping [10]. Text throughout was minimized through use of telegram style and itemized lists (where possible, without bullets).

## Overview of the templates

Below, we present preferred table templates for reporting different types of information in a systematic review of PROMs, along with recommendations on whether to include these in the main manuscript (M) or the supplementary materials (S). The templates serve distinct purposes, from documenting PROM characteristics to reporting on the evaluation of measurement properties:

- Templates for PROM characteristics: these templates structure key characteristics of PROMs, including general characteristics (Template 1, M), interpretability aspects (Template 2, S), and feasibility aspects (Template 3, S).
- Templates for studies’ characteristics: these templates help summarize the characteristics of the studies included in a systematic review of PROMs; one template focusses on PROM development and content validity studies (Template 4, M/S), and one template on the other measurement properties (Template 5, M/S).
- Templates for the evaluation of measurement properties: these templates help to organize the results of the evaluation of measurement properties, including the risk of bias assessment, the evaluation of the individual studies, summarizing the results, and grading the certainty of the evidence; one template can be used for PROM development and content validity studies (Template 6, S), and one template for the other measurement properties (Template 7, S).
- Template for summary of findings: this template presents the summary of findings on the overall evidence for each PROM, including the certainty of the evidence (Template 8, M).

Each template is presented and discussed in detail below. In this communication, we have inserted these templates as figures to preserve formatting. Word files of these templates along with instructions on how to use them are available in the online resources of this article and can be downloaded from www.prisma-cosmin.ca and www.cosmin.nl. Each of the templates contains sample data, resembling the data that need to be reported in systematic reviews of PROMs. For any template, systematic review authors may add rows if more PROMs or studies are included. Authors may, in exceptional cases, add or remove columns applicable to their review, though we would not recommend this. For reviews targeting multidimensional outcomes (e.g., health-related quality of life), we recommend organizing the templates by specific outcomes (e.g., physical functioning, anxiety, fatigue). This reflects the understanding that each subscale of a PROM may have distinct measurement properties and should therefore be treated as a separate PROM [4].

### Templates for PROM characteristics

Most systematic reviews include multiple PROMs. An essential first step is to clearly present the characteristics of each PROM (PRISMA-COSMIN for OMIs 2024 reporting item #23a). Template 1 provides a format for this purpose. If different versions (e.g., long and shorter versions) of a PROM exist, each version requires a separate row. If a multidimensional PROM is included in the review, only the subscale(s) of interest need to be included, and a footnote may be added as was done for the first PROM. If desired, additional columns can be added, for example to list the measurement properties that were evaluated for each PROM. However, this will make the template larger and potentially harder to interpret.

**Table 2:**
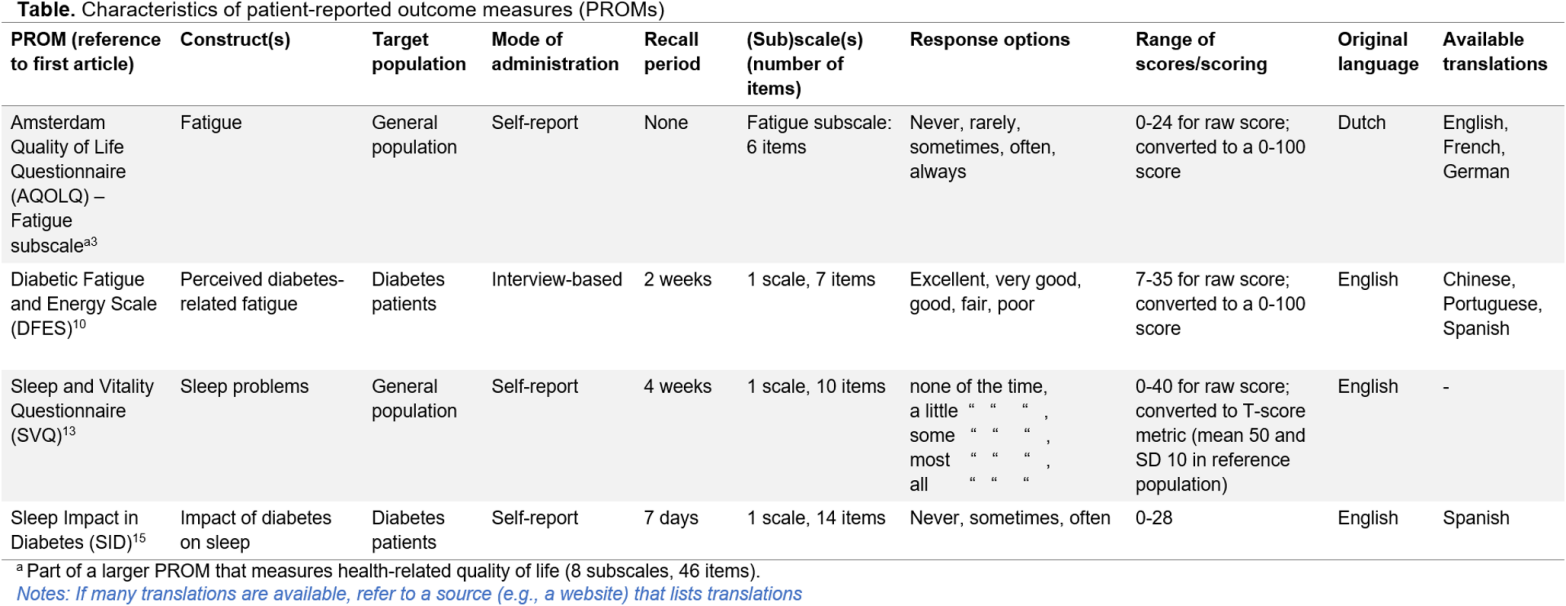
**Template 1.** Template for reporting PROM characteristics – PRISMA-COSMIN for OMIs 2024 Item #23a

Other key PROM characteristics are interpretability and feasibility. Interpretability concerns the qualitative meaning attributed to a quantitative score [15]. Feasibility refers to whether a PROM can be applied easily, given constraints of time, money, and other practical aspects [16, 17]. This is particularly relevant if the review aims to select the most suitable PROM for a particular purpose [4, 9]. Template 2 provides a format for reporting interpretability aspects for each included PROM; Template 3 presents a format for reporting feasibility aspects (PRISMA-COSMIN for OMIs 2024 reporting items #23b and #23c).

**Table 3:**
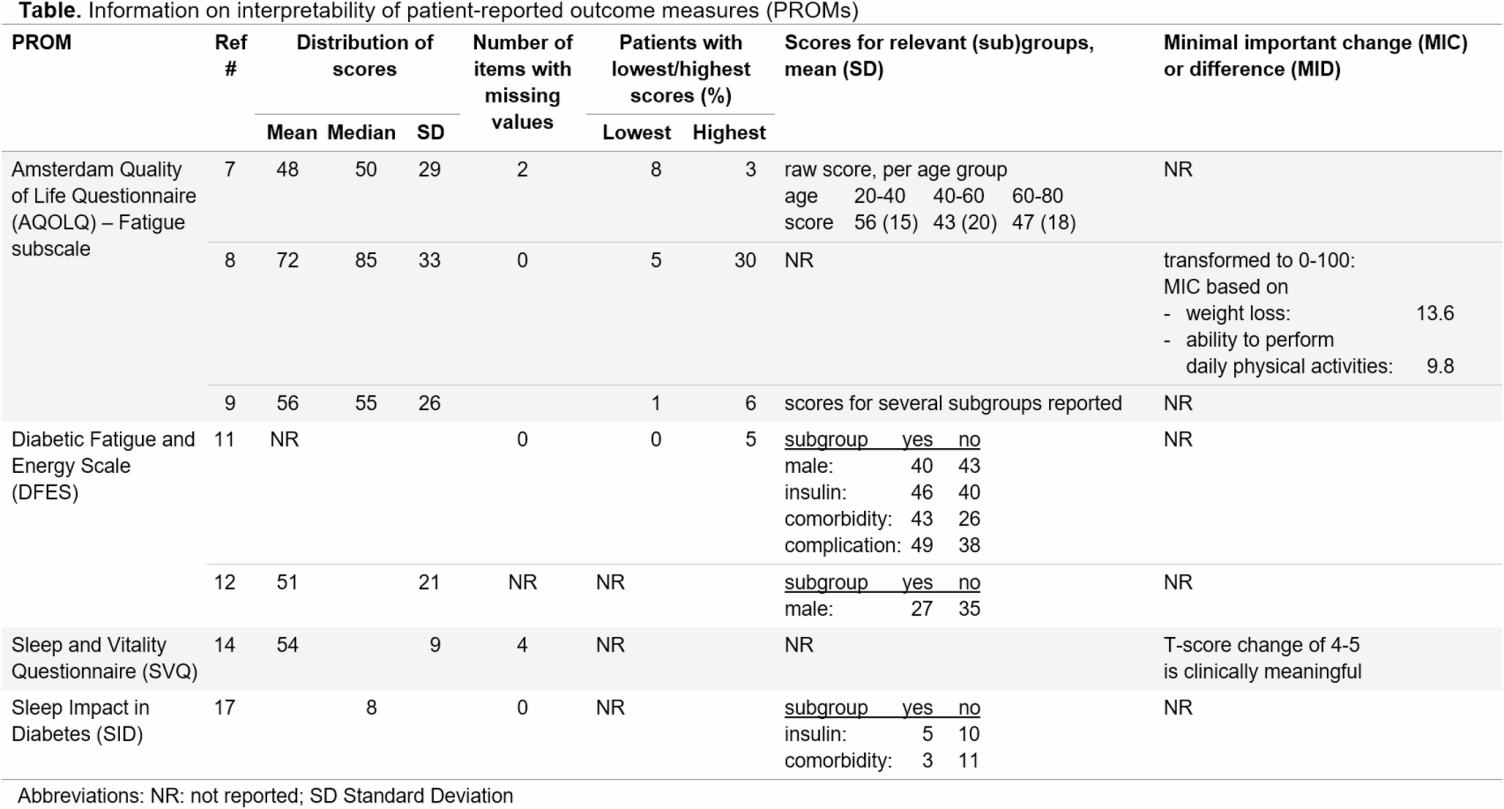
**Template 2. **Template for reporting interpretability aspects of PROMs – PRISMA-COSMIN for OMIs 2024 Item #23b

**Table 4:**
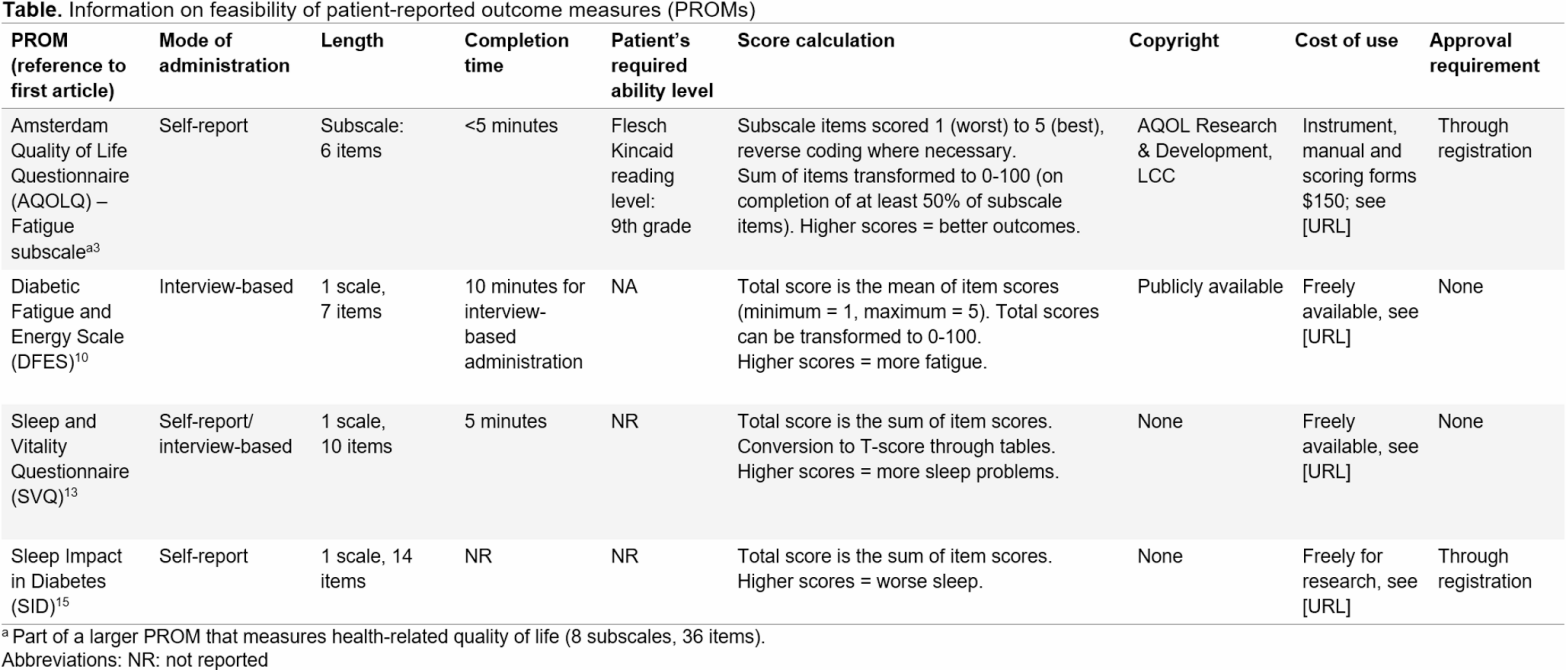
**Template 3. **Template for reporting feasibility aspects of PROMs – PRISMA-COSMIN for OMIs 2024 Item #23c

### Templates for study characteristics

In most systematic reviews, multiple study reports (e.g., articles) are included that provide evidence on the measurement properties of a PROM, often containing multiple studies (i.e., one for each measurement property). Reporting the characteristics of the included studies (PRISMA-COSMIN for OMIs 2024 reporting items #24) allows end-users to understand the generalizability of the results of the included studies, and the applicability of the conclusions and recommendations of the review for their purpose [18]. Template 4 provides a format for presenting the study characteristics of PROM development and content validity studies in a tabular format. In these studies, a distinction between patient and professional input is made, and the type of input provided needs to be reported. Template 5 provides a format for presenting the study characteristics of the remaining measurement properties. This template allows for separate reporting of key characteristics, for example if multiple measurement properties are evaluated in one report, but in different samples. An example of this is presented in the first row of Template 5, where a sub row is created for the characteristics of the reliability study. These tables will often be included in the supplementary materials, although depending on the journal and its readership they could also be included in the main manuscript (see Table 1 for recommended locations).

**Table 5:**
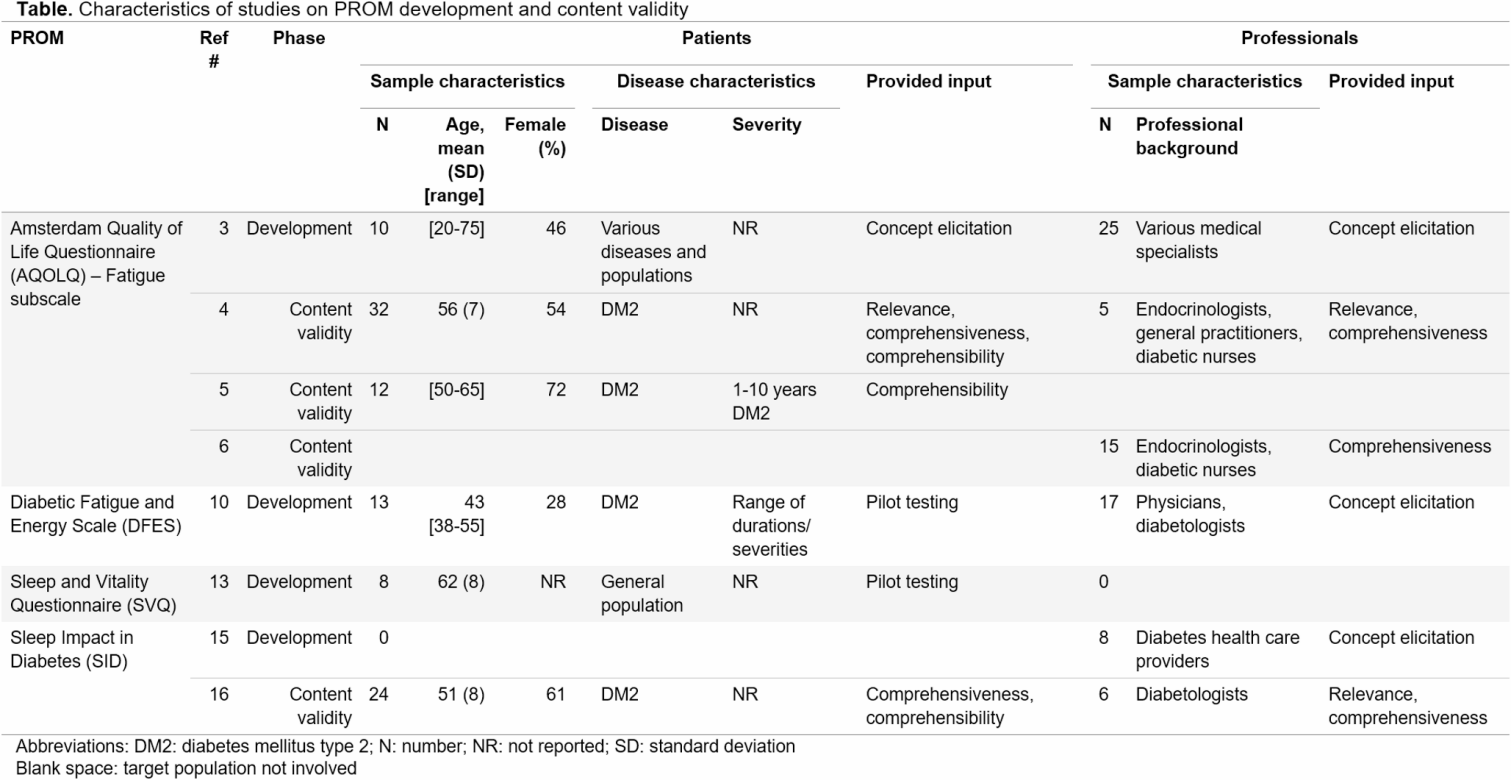
**Template 4. **Template for reporting study characteristics for PROM development and content validity – PRISMA-COSMIN for OMIs 2024 Item #24

**Table 6:**
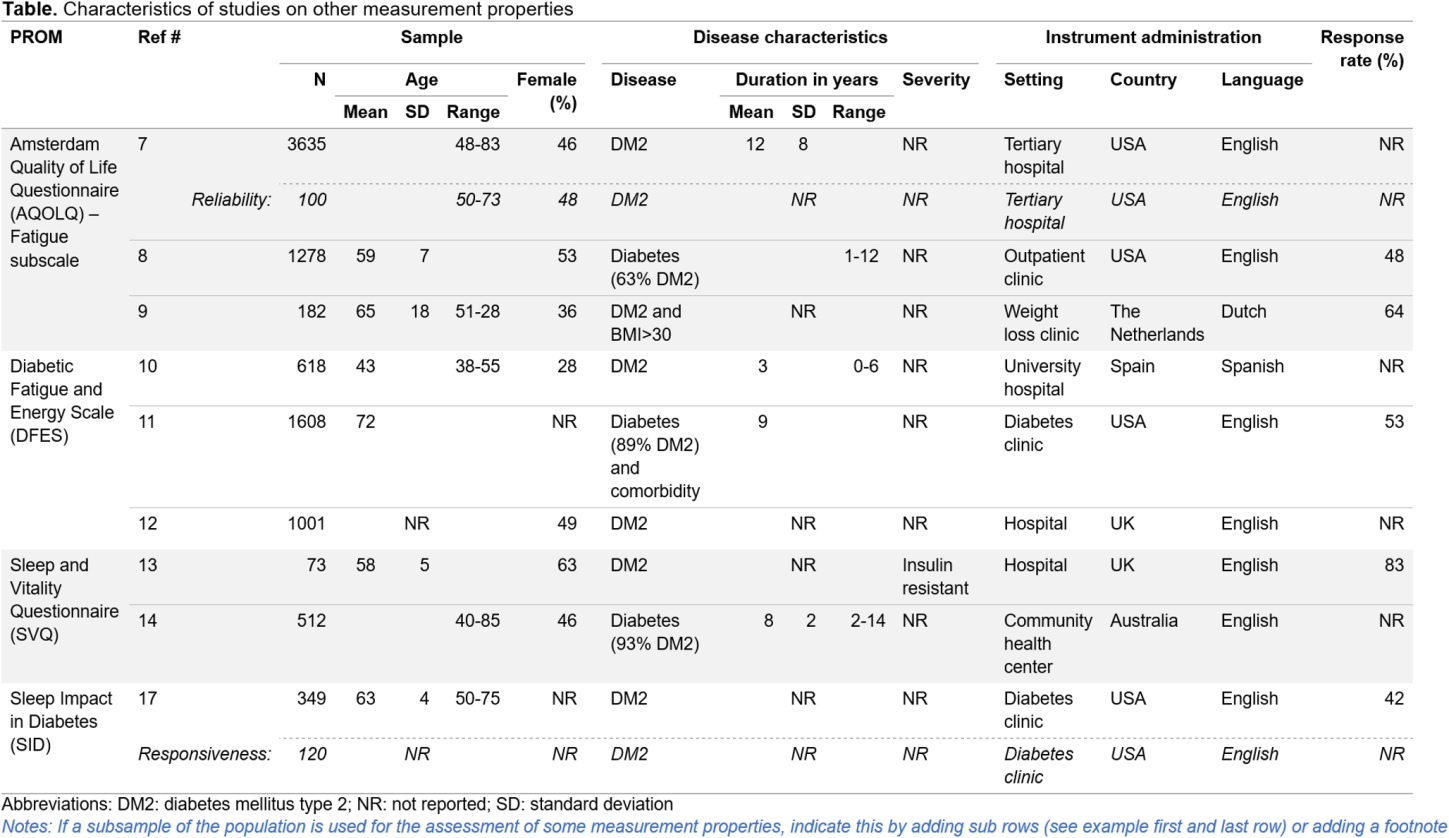
**Template 5. **Template for reporting study characteristics of other measurement properties – PRISMA-COSMIN for OMIs 2024 Item #24

### Templates for the measurement properties of PROMs

The evaluation of measurement properties is the core of a systematic review of PROMs. First, content validity is being evaluated based on PROM development, content validity studies with patients and experts (if any), and a rating of the reviewers themselves [19]. Because the evaluation of content validity results in a different type of data compared to the other measurement properties, a separate template is required. As such, Template 6 provides a format for reporting the results of the risk of bias assessment of the PROM development and content validity studies, as well as content validity ratings for these two phases and the ratings of reviewers. The summarized rating and the certainty of the evidence can be included as well. Comments can be made to explain why one of the three aspects of content validity (i.e., relevance, comprehensiveness and comprehensibility) is not sufficient.

**Table 7:**
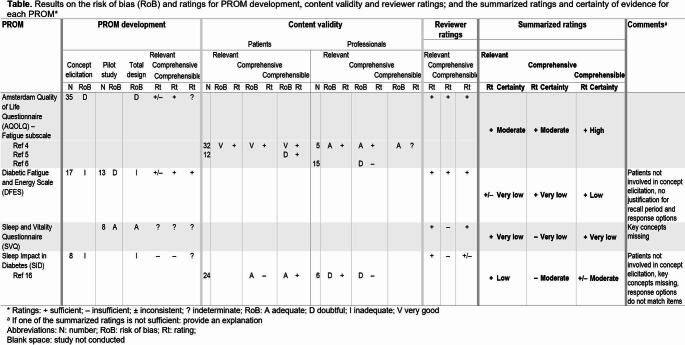
**Template 6. **Template for reporting content validity – PRISMA-COSMIN for OMIs 2024 Item #25, 26, 27a, 28

After the evaluation of content validity, the other measurement properties are being evaluated. First, a risk of bias assessment for each individual study is conducted, to determine whether the results can be trusted [20]. After that, the results on measurement properties) of individual studies are extracted and evaluated against quality criteria (e.g., criteria for good measurement properties) [4]. Then the results of all individual studies are synthesized per measurement property, which means that the results of individual studies for each measurement property are summarized or pooled, and this summarized result is again rated against quality criteria [1, 4]. Next, a certainty assessment is conducted to express confidence in the body of evidence. Template 7 combines all information relevant to the measurement properties of PROMs, both from the evaluation of individual studies, as well as from the evaluation of PROMs. Note that the first four measurement properties are displayed in the top half of the template, and the last four measurement properties are displayed in the bottom half (content validity is reported in Template 6). The template can be repeated over multiple pages if numerous PROMs are included. In that case, the header row should be repeated on every page. In implementation, care must be taken to ensure all footnotes are shown, but only on those pages where they are relevant.

**Table 8:**
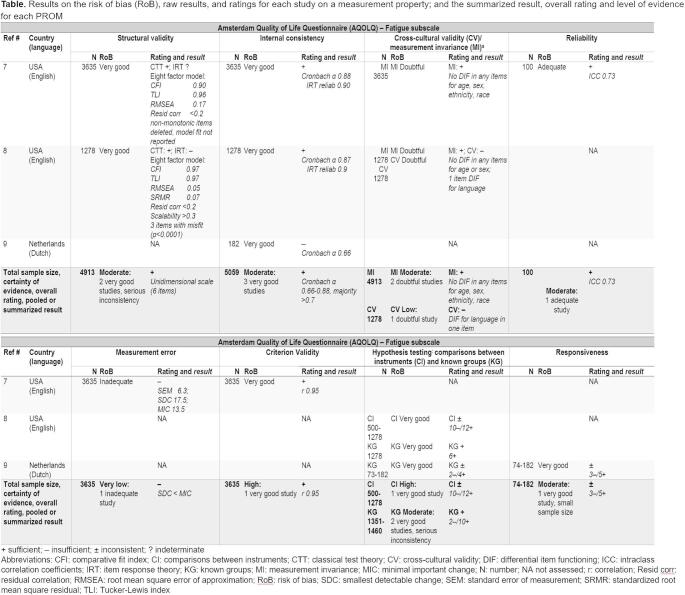
**Template 7. **Template for results of the systematic review – PRISMA-COSMIN for OMIs 2024 Item #25, 26, 27a, 28

### Template for the summary of findings

The most important end product of a systematic review of PROMs is the summary of findings: summarized ratings of the measurement properties along with a grading of the certainty of the evidence. Although these data are included in Template 7, this template does not allow for an easy comparison across PROMs. Template 8 facilitates such comparison. Here, colors are used to reenforce the message and horizontal comparisons are possible because each column represents a different PROM. However, if many PROMs are included, it might not be possible to fit this table on a landscape page. Therefore, a flipped alternative is provided in the online resources (Template 8a).

**Table 9:**
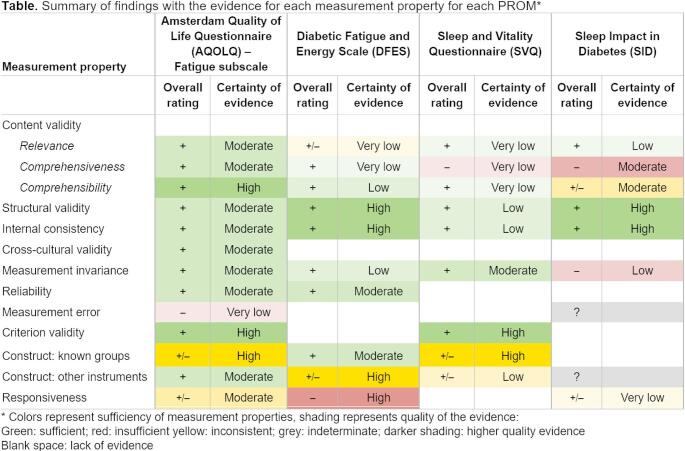
**Template 8. **Template for summary of findings (i.e., overall rating against quality criteria and certainty assessment) – PRISMA-COSMIN for OMIs 2024 Item #27a, 28

## Discussion

We provide eight templates of tables designed to facilitate a clear and structured presentation of results in systematic reviews of OMIs, with a focus on PROMs. These templates are freely available at www.prisma-cosmin.ca and www.cosmin.nl, and in the online resources of this article. The templates can assist authors in preparing the reports of their reviews, potentially saving them a lot of time. The templates also help end-users of OMI systematic reviews, such as researchers, clinicians, core outcome set developers, and regulators, to interpret systematic review findings and to select an appropriate OMI for their specific needs. Together with the previously published *PRISMA-COSMIN for OMIs 2024* flow diagram [9], a template for each main *PRISMA-COSMIN for OMIS 2024* reporting item in the results section is now available.

Although *PRISMA-COSMIN for OMIs 2024* states in its checklist *what* needs to be reported and explains in the Explanation and Elaboration document *why* and *how* to report these items [9], structured table templates were not yet provided. While the COSMIN manual includes some templates, these do not fully adhere to the key design principles of *clear vision* and *clear understanding* [10, 11], limiting their effectiveness in presenting results clearly and consistently. Moreover, while OMERACT has recently developed templates that meet some of our needs [8], these focus on the measurement properties critical for Core Outcome Set development. A recent overview of 100 recently published OMI systematic reviews found large variability in the presentation of results, often leading to suboptimal communication [21]. This makes it difficult for end-users to independently judge the results and select the most appropriate OMI for their specific application. This manuscript outlines manners in which authors can present necessary information in the form of the developed table templates, supporting clear vision and clear understanding. In line with current standards for research transparency, all relevant review data should be made available, whether in the main manuscript, supplementary materials, or public repositories. These templates will enable comprehensive reporting of key systematic review data, facilitating critical appraisal and informed decision-making by end-users.

The eight templates aim to standardize and improve reporting practice, complementing *PRISMA-COSMIN for OMIs 2024* and provide authors with a comprehensive set of tools to effectively report their systematic review of OMIs. The templates effectively combine different *PRISMA-COSMIN for OMIs 2024* reporting items [9], and provide clear recommendations on whether to include these in the main manuscript or the supplementary materials. Similar as to PRISMA-COSMIN for OMIs 2024, the templates can be used for systematic reviews conducted with any methodology. They do not apply specifically to systematic reviews conducted with the methodology or tools from the COSMIN initiative, although it is consistent with COSMIN guidance [1, 4]. While we recognize that journals often have their own typesetting conventions, we believe it is beneficial to present tables of systematic reviews of OMIs in a harmonized, proper format [10]. We emphasize their value in preparing systematic review tables in a clear and consistent format, and suggest that editors follow this by allowing the tables to be published in the submitted format, i.e. as images rather than as tables from a Word processor. But even if modified during the publication process, our format remains valuable in other knowledge translation materials, such as websites, newsletters, and reports.

We encourage authors to customize these templates to fit their specific needs as applicable to their systematic review, such as adding or removing rows. The templates presented here are specifically developed for systematic reviews of PROMs, but should also be useable for systematic reviews of other types of questionnaires, such as clinician-reported outcome measures, observer-reported outcome measures, or patient-reported experience measures (PREMs). For other types of OMIs, such as performance-based tests and clinical examinations, adaptations to the templates may be necessary. If needed, authors can incorporate additional tables (e.g., for measurement property definitions or criteria) using the same style and formatting as used in the provided templates. We suggest systematic review authors to adopt these templates, ultimately fostering greater clarity and consistency in reporting the results of OMI systematic reviews.

## Conclusion

An international group of experts developed eight templates to be used as complement to the reporting items in *PRISMA-COSMIN for OMIs 2024* guidance. The templates align with the latest COSMIN guidelines for conducting systematic reviews of PROMs, and provide authors with a comprehensive set of tools to effectively report their OMI systematic review, and to standardize and improve reporting practice.

## Supplementary Information

Below is the link to the electronic supplementary material.

**Supplementary file 1. **Table templates for the results of systematic reviews of patient-reported outcome measures (PROMs)

**Supplementary file 2. **

## Declarations

### Data availability statement

The data used in the current manuscript are included in the manuscript and in the Supplementary Information.

## References

[1] Prinsen, C. A., Mokkink, L. B., Bouter, L. M., Alonso, J., Patrick, D. L., De Vet, H. C., & Terwee, C. B. (2018). COSMIN guideline for systematic reviews of patient-reported outcome measures. *Quality of Life Research,**27*(5), 1147–1157. 29435801 10.1007/s11136-018-1798-3 PMC5891568

[2] COSMIN. (2022). *COSMIN database of systematic reviews*. www.cosmin.nl/tools/database-systematic-reviews/.

[3] Beaton, D. E., Maxwell, L. J., Shea, B. J., Wells, G. A., Boers, M., Grosskleg, S., Bingham, C. O., Conaghan, P. G., D’agostino, M. A., De Wit, M. P., & Gossec, L. (2019). Instrument selection using the OMERACT filter 2.1: The OMERACT methodology. *The Journal of rheumatology,**46*(8), 1028–1035. 30709952 10.3899/jrheum.181218

[4] Mokkink, L. B., Elsman, E. B., & Terwee, C. B. (2024). COSMIN guideline for systematic reviews of patient-reported outcome measures version 2.0. *Quality of Life Research,**33*(11), 2929–2939. 39198348 10.1007/s11136-024-03761-6 PMC11541334

[5] OMERACT. (2025). *The OMERACT Handbook update*.

[6] Mokkink, L. B., Elsman, E. B. M., & Terwee, C. B. (2024). *Conducting a systematic review of patient-reported outcome measures: manual version 2.0*. COSMIN. 10.1007/s11136-024-03761-6 PMC11541334 39198348

[7] COSMIN. (2024). *COSMIN review management file*.

[8] OMERACT. (2025). *Downloadable Forms* 2025 [cited 2025 July 22]; Available from: https://omeract.org/resources/downloadable-forms/.

[9] Elsman, E. B., Mokkink, L. B., Terwee, C. B., Beaton, D., Gagnier, J. J., Tricco, A. C., Baba, A., Butcher, N. J., Smith, M., Hofstetter, C., & Aiyegbusi, O. L. (2024). Guideline for reporting systematic reviews of outcome measurement instruments (OMIs): PRISMA-COSMIN for OMIs 2024. *Journal of Clinical Epidemiology,**173*, Article 111422. 38849061 10.1016/j.jclinepi.2024.111422

[10] Boers, M. (2022). *Data visualization for biomedical scientists: Creating tables and graphs that work*. VU University Press.

[11] Tufte, E. R., & Graves-Morris, P. R. (1983). *The visual display of quantitative information* (Vol. 2). Graphics Press.

[12] Few, S. (2004). *Show me the numbers.* Analytics Pres, **2**.

[13] Boers, M. (2018). Graphics and statistics for cardiology: Designing effective tables for presentation and publication. *Heart,**104*(3), 192–200. 29030423 10.1136/heartjnl-2017-311581

[14] Beaton, D., Boers, M., Bingham, C. O., Maxwell, L. J., Conaghan, P. G., Grosskleg, S., Hofstetter, C., Shea, B. J., Simon, L., Tugwell, P., & Wells, G. A. (2025). Summary of findings tables for measurement property reviews: The evolution and application of OMERACT’s summary of measurement properties (SOMP) table. *Seminars in Arthritis and Rheumatism,**72*, Article 152664. 40086155 10.1016/j.semarthrit.2025.152664

[15] Mokkink, L. B., Terwee, C. B., Patrick, D. L., Alonso, J., Stratford, P. W., Knol, D. L., Bouter, L. M., & de Vet, H. C. (2010). The COSMIN study reached international consensus on taxonomy, terminology, and definitions of measurement properties for health-related patient-reported outcomes. *Journal of Clinical Epidemiology,**63*(7), 737–745. 20494804 10.1016/j.jclinepi.2010.02.006

[16] Boers, M., Beaton, D. E., Shea, B. J., Maxwell, L. J., Bartlett, S. J., Bingham, C. O., Conaghan, P. G., D’agostino, M. A., De Wit, M. P., Gossec, L., & March, L. (2019). OMERACT Filter 2.1: Elaboration of the conceptual framework for outcome measurement in health intervention studies. *The Journal of rheumatology,**46*(8), 1021–1027. 30770515 10.3899/jrheum.181096

[17] Boers, M., Brooks, P., Strand, C. V., & Tugwell, P. (1998). The OMERACT filter for outcome measures in rheumatology. *The Journal of rheumatology,**25*(2), 198–199. 9489805

[18] Page, M. J., McKenzie, J. E., Bossuyt, P. M., Boutron, I., Hoffmann, T. C., Mulrow, C. D., Shamseer, L., Tetzlaff, J. M., Akl, E. A., Brennan, S. E., Chou, R., & The, P. R. I. S. M. A. (2020). statement: An updated guideline for reporting systematic reviews. *BMJ,**2021*, 372. 10.1136/bmj.n71 PMC8005924 33782057

[19] Terwee, C. B., Prinsen, C. A., Chiarotto, A., Westerman, M. J., Patrick, D. L., Alonso, J., Bouter, L. M., De Vet, H. C., & Mokkink, L. B. (2018). COSMIN methodology for evaluating the content validity of patient-reported outcome measures: A Delphi study. *Quality of Life Research,**27*(5), 1159–1170. 29550964 10.1007/s11136-018-1829-0 PMC5891557

[20] Mokkink, L. B., De Vet, H. C., Prinsen, C. A., Patrick, D. L., Alonso, J., Bouter, L. M., & Terwee, C. B. (2018). COSMIN risk of bias checklist for systematic reviews of patient-reported outcome measures. *Quality of Life Research,**27*, 1171–1179. 29260445 10.1007/s11136-017-1765-4 PMC5891552

[21] Elsman, E. B., Mokkink, L. B., Abma, I. L., Aiyegbusi, O. L., Chiarotto, A., Haywood, K. L., Matvienko-Sikar, K., Oosterveer, D. M., Pool, J. J., Swinkels-Meewisse, I. E., & Offringa, M. (2024). Methodological quality of 100 recent systematic reviews of health-related outcome measurement instruments: An overview of reviews. *Quality of Life Research*. 10.1007/s11136-024-03706-z PMC11452433 38961010

