# Supplementary material for: Systematic reviews of patient-reported outcome measures (PROMs): table templates for effective communication
Source: Qual Life Res. 2025 Sep 3;34(12):3485–95. doi: 10.1007/s11136-025-04058-y (PMC12689657; doi:10.1007/s11136-025-04058-y)
Supplement: Supplementary file 1 — Table templates for the results of systematic reviews of patient-reported outcome measures (PROMs) [file 11136_2025_4058_MOESM1_ESM.pdf]

Systematic reviews of patient-reported outcome measures (PROMs): table templates for effective communication

Elsman EBM, Boers M, Terwee CB, Beaton D, Abma I, Aiyegbusi OL, Chiarotto A, Haywood K, Matvienko-Sikar K, Mehdipour A, Oosterveer DM, Mokkink LB, Offringa M

Online resource 1:

Table templates for the results of systematic reviews of patient-reported outcome measures (PROMs)

How to use these templates:

Eight table templates for reporting different types of information in a systematic review of patient-reported outcome measures (PROMs) are presented. These templates have been carefully designed to support the clear and consistent reporting of data, and complement *PRISMA-COSMIN for OMIs 2024*.[1] The structure, layout, and formatting aim to balance comprehensiveness with legibility. Please follow the guidance below to ensure optimal use.

General use:

- To illustrate how these templates should be structured, each has been populated with sample data; this sample data is intended only to demonstrate layout and organization – it does not represent actual study findings.
- The templates reflect COSMIN guideline for conducting systematic reviews of PROMs,[2] but can also be used for other methodology, although adaptations might be needed.
- While these templates are designed for reviews of PROMs, they may be adapted for reviews of other types of outcome measurement instruments (OMIs).
- Adding, removing or altering the layout of columns is not recommended to preserve formatting and alignment; rows may be added or deleted to reflect the number of PROMs, subscales, or studies.
- Tables should preferably fit on one page. If this is not possible, the header row(s) are formatted to be repeated on subsequent pages. Footnotes should be present on each page where they are relevant. To achieve this, it is best to create a hard split in the final submission format, add/repeat the footnotes and label the tables with 'A', 'B' etc. for the separate pages.
- The templates are best saved and submitted as PDF files to preserve formatting. Proper page width can be assured by decreasing the magnification factor for printing.

Formatting and layout:

- Gridlines and rules:
  - Gridlines are visible for ease of use and can be hidden via the *Table Layout* menu.
  - Tables use very light background shading to emphasize rows and avoid horizontal lines; if rules are used, they are light gray and thin to avoid obscuring content.
- Cell margins: Except for Template 6 and 8, all tables use the following cell margins:
  - Top: 0.1 cm, Bottom: 0.05 cm, Left/Right: 0.15 cm.
    These margins provide sufficient white space for readability while maximizing space efficiency.
- Orientation: Data are presented such to facilitate comparison between PROMs and across studies.
- Shading: Use alternating row shading to distinguish between PROMs.
- Indents: if indents are needed within a cell, use <option-tab> for Mac or <alt-tab> for Windows.

---

[1] Elsman, E.B.M, et al., *Guideline for reporting systematic reviews of outcome measurement instruments (OMIs): PRISMA-COSMIN for OMIs 2024*. Journal of Clinical Epidemiology, 2024.
[2] Mokkink L.B., Elsman, E.B.M., and Terwee, C.B., *COSMIN guideline for systematic reviews of patient-reported outcome measures version 2.0.* Qual Life Res, 2024. **33**(11): p. 2929-2939.

Structure:

- PROM versions and subscales:
  - Each version or subscale of a PROM is considered a separate PROM and should be listed on a separate row.
  - PROMs and subscales should be grouped by outcome or construct.
- Multiple sources per PROM:
  - When multiple study reports exist, list additional studies in rows below.
  - Do not insert multiple citations in a single cell—use separate rows instead.

Text and numerical formatting:

- Punctuation: Use an en dash (–) for legibility.
- Alignment and precision:
  - Text is generally left-aligned; numbers are right-aligned or decimal-aligned.
  - Percentages can be shown as integers; other results can also often be summarized with two significant digits (e.g., 12, 1.2, 0.12) unless greater precision is warranted.
  - Each data point should be placed in its own cell wherever possible.
- Line breaks in text phrases: Use manual line breaks at natural pauses (not automatic word wrapping) to improve readability.
- Concise writing: Use telegram style and itemized lists where possible, omitting unnecessary words and punctuation.

Overview of the templates:

- The eight templates serve distinct purposes, from documenting PROM characteristics to reporting on the evaluation of measurement properties.
- For each template, recommendations on whether to include these in the main manuscript (M) or the supplementary materials (S) are made.
  - Templates for PROM characteristics: these templates structure key characteristics of PROMs, including general characteristics (Template 1, M), interpretability aspects (Template 2, S), and feasibility aspects (Template 3, S).
  - Templates for studies' characteristics: these templates help summarize the characteristics of the studies included in a systematic review of PROMs; one template focusses on PROM development and content validity studies (Template 4, M/S), and one template on the other measurement properties (Template 5, M/S).
  - Templates for the evaluation of measurement properties: these templates help to organize the results of the evaluation of measurement properties, including the risk of bias assessment, the evaluation of the individual studies, summarizing the results, and grading the certainty of the evidence; one template can be used for PROM development and content validity studies (Template 6, S), and one template for the other measurement properties (Template 7, S).
  - Template for summary of findings: this template presents the summary of findings on the overall evidence for each PROM, including the certainty of the evidence (Template 8, M).

Template 1

Table. Characteristics of patient-reported outcome measures (PROMs)

PROM (reference to first article)	Construct(s)	Target population	Mode of administration	Recall period	(Sub)scale(s) (number of items)	Response options	Range of scores/scoring	Original language	Available translations
Amsterdam Quality of Life Questionnaire (AQOLQ) – Fatigue subscale[a3]	Fatigue	General population	Self-report	None	Fatigue subscale: 6 items	Never, rarely, sometimes, often, always	0-24 for raw score; converted to a 0-100 score	Dutch	English, French, German
Diabetic Fatigue and Energy Scale (DFES)[10]	Perceived diabetes-related fatigue	Diabetes patients	Interview-based	2 weeks	1 scale, 7 items	Excellent, very good, good, fair, poor	7-35 for raw score; converted to a 0-100 score	English	Chinese, Portuguese, Spanish
Sleep and Vitality Questionnaire (SVQ)[13]	Sleep problems	General population	Self-report	4 weeks	1 scale, 10 items	none of the time,<br>a little " " " ,<br>some " " " ,<br>most " " " ,<br>all " " "	0-40 for raw score; converted to T-score metric (mean 50 and SD 10 in reference population)	English	-
Sleep Impact in Diabetes (SID)[15]	Impact of diabetes on sleep	Diabetes patients	Self-report	7 days	1 scale, 14 items	Never, sometimes, often	0-28	English	Spanish

[a] Part of a larger PROM that measures health-related quality of life (8 subscales, 46 items).

Notes: If many translations are available, refer to a source (e.g., a website) that lists translations

Template 2

Table. Information on interpretability of patient-reported outcome measures (PROMs)

PROM	Ref #	Distribution of scores			Number of items with missing values	Patients with lowest/highest scores (%)		Scores for relevant (sub)groups, mean (SD)	Minimal important change (MIC) or difference (MID)
		Mean	Median	SD		Lowest	Highest		
Amsterdam Quality of Life Questionnaire (AQOLQ) – Fatigue subscale	7	48	50	29	2	8	3	raw score, per age group<br>age 20-40 40-60 60-80<br>score 56 (15) 43 (20) 47 (18)	NR
	8	72	85	33	0	5	30	NR	transformed to 0-100:<br>MIC based on<br>- weight loss: 13.6<br>- ability to perform daily physical activities: 9.8
	9	56	55	26		1	6	scores for several subgroups reported	NR
Diabetic Fatigue and Energy Scale (DFES)	11	NR			0	0	5	subgroup yes no<br>male: 40 43<br>insulin: 46 40<br>comorbidity: 43 26<br>complication: 49 38	NR
	12	51		21	NR	NR		subgroup yes no<br>male: 27 35	NR
Sleep and Vitality Questionnaire (SVQ)	14	54		9	4	NR		NR	T-score change of 4-5 is clinically meaningful
Sleep Impact in Diabetes (SID)	17		8		0	NR		subgroup yes no<br>insulin: 5 10<br>comorbidity: 3 11	NR

Abbreviations: NR: not reported; SD Standard Deviation

Template 3

Table. Information on feasibility of patient-reported outcome measures (PROMs)

PROM (reference to first article)	Mode of administration	Length	Completion time	Patient's required ability level	Score calculation	Copyright	Cost of use	Approval requirement
Amsterdam Quality of Life Questionnaire (AQOLQ) – Fatigue subscale[a3]	Self-report	Subscale: 6 items	<5 minutes	Flesch Kincaid reading level: 9th grade	Subscale items scored 1 (worst) to 5 (best), reverse coding where necessary. Sum of items transformed to 0-100 (on completion of at least 50% of subscale items). Higher scores = better outcomes.	AQOL Research & Development, LCC	Instrument, manual and scoring forms $150; see [URL]	Through registration
Diabetic Fatigue and Energy Scale (DFES)[10]	Interview-based	1 scale, 7 items	10 minutes for interview-based administration	NA	Total score is the mean of item scores (minimum = 1, maximum = 5). Total scores can be transformed to 0-100. Higher scores = more fatigue.	Publicly available	Freely available, see [URL]	None
Sleep and Vitality Questionnaire (SVQ)[13]	Self-report/ interview-based	1 scale, 10 items	5 minutes	NR	Total score is the sum of item scores. Conversion to T-score through tables. Higher scores = more sleep problems.	None	Freely available, see [URL]	None
Sleep Impact in Diabetes (SID)[15]	Self-report	1 scale, 14 items	NR	NR	Total score is the sum of item scores. Higher scores = worse sleep.	None	Freely for research, see [URL]	Through registration

[a] Part of a larger PROM that measures health-related quality of life (8 subscales, 36 items).
Abbreviations: NR: not reported

Template 4

Table. Characteristics of studies on PROM development and content validity

PROM	Ref #	Phase	Patients						Professionals		
			Sample characteristics			Disease characteristics		Provided input	Sample characteristics		Provided input
			N	Age, mean (SD) [range]	Female (%)	Disease	Severity		N	Professional background	
Amsterdam Quality of Life Questionnaire (AQOLQ) – Fatigue subscale	3	Development	10	[20-75]	46	Various diseases and populations	NR	Concept elicitation	25	Various medical specialists	Concept elicitation
	4	Content validity	32	56 (7)	54	DM2	NR	Relevance, comprehensiveness, comprehensibility	5	Endocrinologists, general practitioners, diabetic nurses	Relevance, comprehensiveness
	5	Content validity	12	[50-65]	72	DM2	1-10 years DM2	Comprehensibility			
	6	Content validity							15	Endocrinologists, diabetic nurses	Comprehensiveness
Diabetic Fatigue and Energy Scale (DFES)	10	Development	13	43 [38-55]	28	DM2	Range of durations/ severities	Pilot testing	17	Physicians, diabetologists	Concept elicitation
Sleep and Vitality Questionnaire (SVQ)	13	Development	8	62 (8)	NR	General population	NR	Pilot testing	0		
Sleep Impact in Diabetes (SID)	15	Development	0						8	Diabetes health care providers	Concept elicitation
	16	Content validity	24	51 (8)	61	DM2	NR	Comprehensiveness, comprehensibility	6	Diabetologists	Relevance, comprehensiveness

Abbreviations: DM2: diabetes mellitus type 2; N: number; NR: not reported; SD: standard deviation
Blank space: target population not involved

Template 5

Table. Characteristics of studies on other measurement properties

PROM	Ref #	Sample					Disease characteristics					Instrument administration			Response rate (%)
		N	Age			Female (%)	Disease	Duration in years			Severity	Setting	Country	Language	
			Mean	SD	Range			Mean	SD	Range					
Amsterdam Quality of Life Questionnaire (AQOLQ) – Fatigue subscale	7	3635			48-83	46	DM2	12	8		NR	Tertiary hospital	USA	English	NR
	Reliability:	*100*			*50-73*	*48*	*DM2*		*NR*		*NR*	*Tertiary hospital*	*USA*	*English*	*NR*
	8	1278	59	7		53	Diabetes (63% DM2)			1-12	NR	Outpatient clinic	USA	English	48
	9	182	65	18	51-28	36	DM2 and BMI>30		NR		NR	Weight loss clinic	The Netherlands	Dutch	64
Diabetic Fatigue and Energy Scale (DFES)	10	618	43		38-55	28	DM2	3		0-6	NR	University hospital	Spain	Spanish	NR
	11	1608	72			NR	Diabetes (89% DM2) and comorbidity	9			NR	Diabetes clinic	USA	English	53
	12	1001		NR		49	DM2		NR		NR	Hospital	UK	English	NR
Sleep and Vitality Questionnaire (SVQ)	13	73	58	5		63	DM2		NR		Insulin resistant	Hospital	UK	English	83
	14	512			40-85	46	Diabetes (93% DM2)	8	2	2-14	NR	Community health center	Australia	English	NR
Sleep Impact in Diabetes (SID)	17	349	63	4	50-75	NR	DM2		NR		NR	Diabetes clinic	USA	English	42
	Responsiveness:	*120*		*NR*		*NR*	*DM2*		*NR*		*NR*	*Diabetes clinic*	*USA*	*English*	*NR*

Abbreviations: DM2: diabetes mellitus type 2; NR: not reported; SD: standard deviation

Notes: If a subsample of the population is used for the assessment of some measurement properties, indicate this by adding sub rows (see example first and last row) or adding a footnote

Template 6

Table. Results on the risk of bias (RoB) and ratings for PROM development, content validity and reviewer ratings; and the summarized ratings and certainty of evidence for each PROM*

PROM	PROM development								Content validity														Reviewer ratings			Summarized ratings						Comments[a]
									Patients							Professionals																
	Concept elicitation		Pilot study		Total design	Relevant	Comprehensive	Comprehensible		Relevant		Comprehensive		Comprehensible			Relevant		Comprehensive		Comprehensible		Relevant	Comprehensive	Comprehensible	Relevant		Comprehensive		Comprehensible		
	N	RoB	N	RoB	RoB	Rt	Rt	Rt	N	RoB	Rt	RoB	Rt	RoB	Rt	N	RoB	Rt	RoB	Rt	RoB	Rt	Rt	Rt	Rt	Rt	Certainty	Rt	Certainty	Rt	Certainty	
Amsterdam Quality of Life Questionnaire (AQOLQ) – Fatigue subscale	35	D			D	+/–	+	?															+	+	+	+	Moderate	+	Moderate	+	High	
Ref 4									32	V	+	V	+	V	+	5	A	+	A	+	A	?										
Ref 5									12					D	+																	
Ref 6																15			D	–												
Diabetic Fatigue and Energy Scale (DFES)	17	I	13	D	I	+/–	+	+															+	+	+	+/–	Very low	+	Very low	+	Low	Patients not involved in concept elicitation, no justification for recall period and response options
Sleep and Vitality Questionnaire (SVQ)			8	A	A	?	?	?															+	–	+	+	Very low	–	Very low	+	Very low	Key concepts missing
Sleep Impact in Diabetes (SID)	8	I			I	–	–	?															+	–	+/–	+	Low	–	Moderate	+/–	Moderate	Patients not involved in concept elicitation, key concepts missing, response options do not match items
Ref 16									24			A	–	A	+	6	D	+	D	–												

* Ratings: + sufficient; – insufficient; ± inconsistent; ? indeterminate; RoB: A adequate; D doubtful; I inadequate; V very good

[a] If one of the summarized ratings is not sufficient: provide an explanation

Abbreviations: N: number; RoB: risk of bias; Rt: rating;

Blank space: study not conducted

Template 7

Note: this template is intentionally split 'in the middle' over 2 pages

Table. Results on the risk of bias (RoB), raw results, and ratings for each study on a measurement property; and the summarized result, overall rating and level of evidence for each PROM

Amsterdam Quality of Life Questionnaire (AQOLQ) – Fatigue subscale													
**Ref #**	**Country (language)**	**Structural validity**			**Internal consistency**			**Cross-cultural validity (CV)/ measurement invariance (MI)[a]**			**Reliability**		
		**N**	**RoB**	**Rating and *result***	**N**	**RoB**	**Rating and *result***	**N**	**RoB**	**Rating and *result***	**N**	**RoB**	**Rating and *result***
7	USA (English)	3635	Very good	CTT +; IRT ?<br>Eight factor model:<br>*CFI 0.90*<br>*TLI 0.96*<br>*RMSEA 0.17*<br>*Resid corr <0.2*<br>*non-monotonic items deleted, model fit not reported*	3635	Very good	+<br>*Cronbach α 0.88*<br>*IRT reliab 0.90*	MI 3635	MI Doubtful	MI: +<br>*No DIF in any items for age, sex, ethnicity, race*	100	Adequate	+<br>*ICC 0.73*
8	USA (English)	1278	Very good	CTT: +; IRT: –<br>Eight factor model:<br>*CFI 0.97*<br>*TLI 0.97*<br>*RMSEA 0.05*<br>*SRMR 0.07*<br>*Resid corr <0.2*<br>*Scalability >0.3*<br>*3 items with misfit (p<0.0001)*	1278	Very good	+<br>*Cronbach α 0.87*<br>*IRT reliab 0.9*	MI 1278<br>CV 1278	MI Doubtful<br>CV Doubtful	MI: +; CV: –<br>*No DIF in any items for age or sex;*<br>*1 item DIF for language*			NA
9	Netherlands (Dutch)			NA	182	Very good	–<br>*Cronbach α 0.66*			NA			NA
**Total sample size, certainty of evidence, overall rating, pooled or summarized result**		**4913**	**Moderate:**<br>2 very good studies, serious inconsistency	**+**<br>*Unidimensional scale (6 items)*	**5059**	**Moderate:**<br>3 very good studies	**+**<br>*Cronbach α 0.66-0.88, majority >0.7*	**MI 4913**<br>**CV 1278**	**MI Moderate:**<br>2 doubtful studies<br>**CV Low:**<br>1 doubtful study	**MI: +**<br>*No DIF in any items for age, sex, ethnicity, race*<br>**CV: –**<br>*DIF for language in one item*	**100**	**Moderate:**<br>1 adequate study	**+**<br>*ICC 0.73*

+ sufficient; – insufficient; ± inconsistent; ? indeterminate

Abbreviations: CFI: comparative fit index; CTT: classical test theory; CV: cross-cultural validity; DIF: differential item functioning; ICC: intraclass correlation coefficients; IRT: item response theory; MI: measurement invariance; N: number; NA not assessed; Resid corr: residual correlation; RMSEA: root mean square error of approximation; RoB: risk of bias; SRMR: standardized root mean square residual; TLI: Tucker-Lewis index

Amsterdam Quality of Life Questionnaire (AQOLQ) – Fatigue subscale													
**Ref #**	**Country (language)**	**Measurement error**			**Criterion Validity**			**Hypothesis testing: comparisons between instruments (CI) and known groups (KG)**			**Responsiveness**		
		**N**	**RoB**	**Rating and *result***	**N**	**RoB**	**Rating and *result***	**N**	**RoB**	**Rating and *result***	**N**	**RoB**	**Rating and *result***
7	USA (English)	3635	Inadequate	– *SEM 6.3; SDC 17.5; MIC 13.5*	3635	Very good	+ *r 0.95*			NA			NA
8	USA (English)			NA			NA	CI 500-1278<br>KG 1278	CI Very good<br>KG Very good	CI ± *10–/12+*<br>KG + *6+*			NA
9	Netherlands (Dutch)			NA			NA	KG 73-182	KG Very good	KG ± *2–/4+*	74-182	Very good	± *3–/5+*
**Total sample size, certainty of evidence, overall rating, pooled or summarized result**		**3635**	**Very low:** 1 inadequate study	**–** *SDC < MIC*	**3635**	**High:** 1 very good study	**+** *r 0.95*	**CI 500-1278**<br>**KG 1351-1460**	**CI High:** 1 very good study<br>**KG Moderate:** 2 very good studies, serious inconsistency	**CI ±** *10–/12+*<br>**KG +** *2–/10+*	**74-182**	**Moderate:** 1 very good study, small sample size	**±** *3–/5+*

+ sufficient; – insufficient; ± inconsistent; ? indeterminate

Abbreviations: CI: comparisons between instruments; KG: known groups; MIC: minimal important change; N: number; NA not assessed; r: correlation; RoB: risk of bias; SDC: smallest detectable change; SEM: standard error of measurement

Template 8

Table. Summary of findings with the evidence for each measurement property for each PROM*

Measurement property	Amsterdam Quality of Life Questionnaire (AQOLQ) – Fatigue subscale		Diabetic Fatigue and Energy Scale (DFES)		Sleep and Vitality Questionnaire (SVQ)		Sleep Impact in Diabetes (SID)	
	**Overall rating**	**Certainty of evidence**	**Overall rating**	**Certainty of evidence**	**Overall rating**	**Certainty of evidence**	**Overall rating**	**Certainty of evidence**
Content validity								
Relevance	+	Moderate	+/–	Very low	+	Very low	+	Low
Comprehensiveness	+	Moderate	+	Very low	–	Very low	–	Moderate
Comprehensibility	+	High	+	Low	+	Very low	+/–	Moderate
Structural validity	+	Moderate	+	High	+	Low	+	High
Internal consistency	+	Moderate	+	High	+	Low	+	High
Cross-cultural validity	+	Moderate						
Measurement invariance	+	Moderate	+	Low	+	Moderate	–	Low
Reliability	+	Moderate	+	Moderate				
Measurement error	–	Very low					?	
Criterion validity	+	High			+	High		
Construct: known groups	+/–	High	+	Moderate	+/–	High		
Construct: other instruments	+	Moderate	+/–	High	+/–	Low	?	
Responsiveness	+/–	Moderate	–	High			+/–	Very low

* Colors represent sufficiency of measurement properties, shading represents quality of the evidence:
Green: sufficient; red: insufficient yellow: inconsistent; grey: indeterminate; darker shading: higher quality evidence
Blank space: lack of evidence

Template 8a – alternative

Table. Summary of findings with the evidence for each measurement property for each PROM*

Name PROM	Ratings	Content validity: *Overall*	Content validity: *Relevance*	Content validity: *Comprehensiveness*	Content validity: *Comprehensibility*	Structural validity	Internal consistency	Cross-cultural validity	Measurement invariance	Reliability	Measurement error	Criterion validity	Construct validity: *Known groups*	Construct validity: *Other instruments*	Responsiveness
**Amsterdam Quality of Life Questionnaire (AQOLQ) – Fatigue subscale**	**Overall**		+	+	+	+	+	+	+	+	–	+	+/–	+	+/–
	**Certainty**		Moderate	Moderate	High	Moderate	Moderate	Moderate	Moderate	Moderate	Very low	High	High	Moderate	Moderate
**Diabetic Fatigue and Energy Scale (DFES)**	**Overall**		+/–	+	+	+	+		+	+			+	+/–	–
	**Certainty**		Moderate	Very low	Low	High	High		Low	Moderate			Moderate	High	High
**Sleep and Vitality Questionnaire (SVQ)**	**Overall**		+	–	+	+	+		+			+	+/–	+/–	
	**Certainty**		Very low	Very low	Very low	Low	Low		Moderate			High	High	Low	
**Sleep Impact in Diabetes (SID)**	**Overall**		+	–	+/–	+	+		–		?			?	+/–
	**Certainty**		Very low	Moderate	Moderate	High	High		Low						Very low

* Colors represent sufficiency of measurement properties, shading represents quality of the evidence:
Green: sufficient; red: insufficient yellow: inconsistent; grey: indeterminate; darker shading: higher quality evidence
Blank space: lack of evidence
