# Supplementary material for: Systematic reviews of patient-reported outcome measures (PROMs): table templates for effective communication
Source: Qual Life Res. 2025 Sep 3;34(12):3485–95. doi: 10.1007/s11136-025-04058-y (PMC12689657; doi:10.1007/s11136-025-04058-y)
Supplement: Supplementary file 2 [file 11136_2025_4058_MOESM2_ESM.docx]

**Online resource 1:
Table templates for the results of systematic reviews of patient-reported outcome measures (PROMs)**

**How to use these templates:**

Eight table templates for reporting different types of information in a systematic review of patient-reported outcome measures (PROMs) are presented. These templates have been carefully designed to support the clear and consistent reporting of data, and complement *PRISMA-COSMIN for OMIs 2024*.^[[1]](#footnote-1)^ The structure, layout, and formatting aim to balance comprehensiveness with legibility. Please follow the guidance below to ensure optimal use.

General use:

- To illustrate how these templates should be structured, each has been populated with sample data; this sample data is intended only to demonstrate layout and organization – it does not represent actual study findings.
- The templates reflect COSMIN guideline for conducting systematic reviews of PROMs,^[[2]](#footnote-2)^ but can also be used for other methodology, although adaptations might be needed.
- While these templates are designed for reviews of PROMs, they may be adapted for reviews of other types of outcome measurement instruments (OMIs).
- Adding, removing or altering the layout of columns is not recommended to preserve formatting and alignment; rows may be added or deleted to reflect the number of PROMs, subscales, or studies.
- Tables should preferably fit on one page. If this is not possible, the header row(s) are formatted to be repeated on subsequent pages.
  Footnotes should be present on each page where they are relevant. To achieve this, it is best to create a hard split in the final submission format, add/repeat the footnotes and label the tables with ‘A’, ‘B’ etc. for the separate pages.
- The templates are best saved and submitted as PDF files to preserve formatting. Proper page width can be assured by decreasing the magnification factor for printing.

Formatting and layout:

- Gridlines and rules:
  - Gridlines are visible for ease of use and can be hidden via the *Table Layout* menu.
  - Tables use very light background shading to emphasize rows and avoid horizontal lines; if rules are used, they are light gray and thin to avoid obscuring content.
- Cell margins: Except for Template 6 and 8, all tables use the following cell margins:
  - Top: 0.1 cm, Bottom: 0.05 cm, Left/Right: 0.15 cm.
    These margins provide sufficient white space for readability while maximizing space efficiency.
- Orientation: Data are presented such to facilitate comparison between PROMs and across studies.
- Shading: Use alternating row shading to distinguish between PROMs.
- Indents: if indents are needed within a cell, use <option-tab> for Mac or <alt-tab> for Windows.

Structure:

- PROM versions and subscales:
  - Each version or subscale of a PROM is considered a separate PROM and should be listed on a separate row.
  - PROMs and subscales should be grouped by outcome or construct.
- Multiple sources per PROM:
  - When multiple study reports exist, list additional studies in rows below.
  - Do not insert multiple citations in a single cell—use separate rows instead.

Text and numerical formatting:

- Punctuation: Use an en dash (–) for legibility.
- Alignment and precision:
  - Text is generally left-aligned; numbers are right-aligned or decimal-aligned.
  - Percentages can be shown as integers; other results can also often be summarized with two significant digits (e.g., 12, 1.2, 0.12) unless greater precision is warranted.
  - Each data point should be placed in its own cell wherever possible.
- Line breaks in text phrases: Use manual line breaks at natural pauses (not automatic word wrapping) to improve readability.
- Concise writing: Use telegram style and itemized lists where possible, omitting unnecessary words and punctuation.

Overview of the templates:

- The eight templates serve distinct purposes, from documenting PROM characteristics to reporting on the evaluation of measurement properties.
- For each template, recommendations on whether to include these in the main manuscript (M) or the supplementary materials (S) are made.
  - Templates for PROM characteristics: these templates structure key characteristics of PROMs, including general characteristics (Template 1, M), interpretability aspects (Template 2, S), and feasibility aspects (Template 3, S).
  - Templates for studies’ characteristics: these templates help summarize the characteristics of the studies included in a systematic review of PROMs; one template focusses on PROM development and content validity studies (Template 4, M/S), and one template on the other measurement properties (Template 5, M/S).
  - Templates for the evaluation of measurement properties: these templates help to organize the results of the evaluation of measurement properties, including the risk of bias assessment, the evaluation of the individual studies, summarizing the results, and grading the certainty of the evidence; one template can be used for PROM development and content validity studies (Template 6, S), and one template for the other measurement properties (Template 7, S).
  - Template for summary of findings: this template presents the summary of findings on the overall evidence for each PROM, including the certainty of the evidence (Template 8, M).

# **Template 1**

**Table.** Characteristics of patient-reported outcome measures (PROMs)

| **PROM (reference to first article)** | **Construct(s)** | **Target population** | **Mode of administration** | **Recall period** | **(Sub)scale(s) (number of items)** | **Response options** | **Range of scores/scoring** | **Original language** | **Available translations** |
| --- | --- | --- | --- | --- | --- | --- | --- | --- | --- |
| Amsterdam Quality of Life Questionnaire (AQOLQ) – Fatigue subscale^a3^ | Fatigue | General population | Self-report | None | Fatigue subscale:  6 items | Never, rarely, sometimes, often, always | 0-24 for raw score; converted to a 0-100 score | Dutch | English, French, German |
| Diabetic Fatigue and Energy Scale (DFES)^10^ | Perceived diabetes-related fatigue | Diabetes patients | Interview-based | 2 weeks | 1 scale, 7 items | Excellent, very good, good, fair, poor | 7-35 for raw score; converted to a 0-100 score | English | Chinese, Portuguese, Spanish |
| Sleep and Vitality Questionnaire (SVQ)^13^ | Sleep problems | General population | Self-report | 4 weeks | 1 scale, 10 items | none of the time,  a little “ “ “ ,  some “ “ “ ,  most “ “ “ ,  all “ “ “ | 0-40 for raw score; converted to T-score metric (mean 50 and SD 10 in reference population) | English | - |
| Sleep Impact in Diabetes (SID)^15^ | Impact of diabetes on sleep | Diabetes patients | Self-report | 7 days | 1 scale, 14 items | Never, sometimes, often | 0-28 | English | Spanish |

^a^ Part of a larger PROM that measures health-related quality of life (8 subscales, 46 items).

*Notes: If many translations are available, refer to a source (e.g., a website) that lists translations*

# **Template 2**

**Table.** Information on interpretability of patient-reported outcome measures (PROMs)

| **PROM** | **Ref #** | **Distribution of scores** | | | **Number of items with missing values** | **Patients with lowest/highest scores (%)** | | **Scores for relevant (sub)groups,**  **mean (SD)** | **Minimal important change (MIC)  or difference (MID)** |
| --- | --- | --- | --- | --- | --- | --- | --- | --- | --- |
|  |  | **Mean** | **Median** | **SD** |  | **Lowest** | **Highest** |  |  |
| Amsterdam Quality of Life Questionnaire (AQOLQ) – Fatigue subscale | 7 | 48 | 50 | 29 | 2 | 8 | 3 | raw score, per age group  age 20-40 40-60 60-80  score 56 (15) 43 (20) 47 (18) | NR |
|  | 8 | 72 | 85 | 33 | 0 | 5 | 30 | NR | transformed to 0-100:  MIC based on  - weight loss: 13.6  - ability to perform  daily physical activities: 9.8 |
|  | 9 | 56 | 55 | 26 |  | 1 | 6 | scores for several subgroups reported | NR |
| Diabetic Fatigue and Energy Scale (DFES) | 11 | NR |  |  | 0 | 0 | 5 | subgroup yes no  male: 40 43  insulin: 46 40  comorbidity: 43 26  complication: 49 38 | NR |
|  | 12 | 51 |  | 21 | NR | NR |  | subgroup yes no  male: 27 35 | NR |
| Sleep and Vitality Questionnaire (SVQ) | 14 | 54 |  | 9 | 4 | NR |  | NR | T-score change of 4-5  is clinically meaningful |
| Sleep Impact in Diabetes (SID) | 17 |  | 8 |  | 0 | NR |  | subgroup yes no  insulin: 5 10  comorbidity: 3 11 | NR |

## Abbreviations: NR: not reported; SD Standard Deviation

# **Template 3**

**Table.** Information on feasibility of patient-reported outcome measures (PROMs)

| **PROM (reference to first article)** | **Mode of administration** | **Length** | **Completion time** | **Patient’s required ability level** | **Score calculation** | **Copyright** | | **Cost of use** | | **Approval requirement** | |
| --- | --- | --- | --- | --- | --- | --- | --- | --- | --- | --- | --- |
| Amsterdam Quality of Life Questionnaire (AQOLQ) – Fatigue subscale^a3^ | Self-report | Subscale:  6 items | <5 minutes | Flesch Kincaid reading level:  9th grade | Subscale items scored 1 (worst) to 5 (best), reverse coding where necessary.  Sum of items transformed to 0-100 (on completion of at least 50% of subscale items). Higher scores = better outcomes. | | AQOL Research & Development, LCC | | Instrument, manual and scoring forms $150; see [URL] | | Through registration |
| Diabetic Fatigue and Energy Scale (DFES)^10^ | Interview-based | 1 scale,  7 items | 10 minutes for interview-based administration | NA | Total score is the mean of item scores (minimum = 1, maximum = 5). Total scores can be transformed to 0-100.  Higher scores = more fatigue. | | Publicly available | | Freely available, see [URL] | | None |
| Sleep and Vitality Questionnaire (SVQ)^13^ | Self-report/  interview-based | 1 scale,  10 items | 5 minutes | NR | Total score is the sum of item scores. Conversion to T-score through tables.  Higher scores = more sleep problems. | | None | | Freely available, see [URL] | | None |
| Sleep Impact in Diabetes (SID)^15^ | Self-report | 1 scale, 14 items | NR | NR | Total score is the sum of item scores. Higher scores = worse sleep. | | None | | Freely for research, see [URL] | | Through registration |

^a^ Part of a larger PROM that measures health-related quality of life (8 subscales, 36 items).

Abbreviations: NR: not reported

# **Template 4**

**Table.** Characteristics of studies on PROM development and content validity

| **PROM** | **Ref #** | **Phase** | **Patients** | | | | | | | |  | **Professionals** | | |
| --- | --- | --- | --- | --- | --- | --- | --- | --- | --- | --- | --- | --- | --- | --- |
|  |  |  | **Sample characteristics** | | |  | **Disease characteristics** | |  | **Provided input** |  | **Sample characteristics** | | **Provided input** |
|  |  |  | **N** | **Age, mean (SD) [range]** | **Female (%)** |  | **Disease** | **Severity** |  |  |  | **N** | **Professional background** |  |
| Amsterdam Quality of Life Questionnaire (AQOLQ) – Fatigue subscale | 3 | Development | 10 | [20-75] | 46 |  | Various diseases and populations | NR |  | Concept elicitation |  | 25 | Various medical specialists | Concept elicitation |
|  | 4 | Content validity | 32 | 56 (7) | 54 |  | DM2 | NR |  | Relevance, comprehensiveness, comprehensibility |  | 5 | Endocrinologists, general practitioners, diabetic nurses | Relevance, comprehensiveness |
|  | 5 | Content validity | 12 | [50-65] | 72 |  | DM2 | 1-10 years DM2 |  | Comprehensibility |  |  |  |  |
|  | 6 | Content validity |  |  |  |  |  |  |  |  |  | 15 | Endocrinologists, diabetic nurses | Comprehensiveness |
| Diabetic Fatigue and Energy Scale (DFES) | 10 | Development | 13 | 43  [38-55] | 28 |  | DM2 | Range of durations/ severities |  | Pilot testing |  | 17 | Physicians, diabetologists | Concept elicitation |
| Sleep and Vitality Questionnaire (SVQ) | 13 | Development | 8 | 62 (8) | NR |  | General population | NR |  | Pilot testing |  | 0 |  |  |
| Sleep Impact in Diabetes (SID) | 15 | Development | 0 |  |  |  |  |  |  |  |  | 8 | Diabetes health care providers | Concept elicitation |
|  | 16 | Content validity | 24 | 51 (8) | 61 |  | DM2 | NR |  | Comprehensiveness, comprehensibility |  | 6 | Diabetologists | Relevance, comprehensiveness |

Abbreviations: DM2: diabetes mellitus type 2; N: number; NR: not reported; SD: standard deviation

Blank space: target population not involved

# **Template 5**

**Table.** Characteristics of studies on other measurement properties

| **PROM** | **Ref #** | **Sample** | | | | |  | **Disease characteristics** | | | | |  | **Instrument administration** | | | **Response rate (%)** |
| --- | --- | --- | --- | --- | --- | --- | --- | --- | --- | --- | --- | --- | --- | --- | --- | --- | --- |
|  |  | **N** | **Age** | | | **Female (%)** |  | **Disease** | **Duration in years** | | | **Severity** |  | **Setting** | **Country** | **Language** |  |
|  |  |  | **Mean** | **SD** | **Range** |  |  |  | **Mean** | **SD** | **Range** |  |  |  |  |  |  |
| Amsterdam Quality of Life Questionnaire (AQOLQ) – Fatigue subscale | 7 | 3635 |  |  | 48-83 | 46 |  | DM2 | 12 | 8 |  | NR |  | Tertiary hospital | USA | English | NR |
|  | *Reliability:* | *100* |  |  | *50-73* | *48* |  | *DM2* |  | *NR* |  | *NR* |  | *Tertiary hospital* | *USA* | *English* | *NR* |
|  | 8 | 1278 | 59 | 7 |  | 53 |  | Diabetes  (63% DM2) |  |  | 1-12 | NR |  | Outpatient clinic | USA | English | 48 |
|  | 9 | 182 | 65 | 18 | 51-28 | 36 |  | DM2 and BMI>30 |  | NR |  | NR |  | Weight loss clinic | The Netherlands | Dutch | 64 |
| Diabetic Fatigue and Energy Scale (DFES) | 10 | 618 | 43 |  | 38-55 | 28 |  | DM2 | 3 |  | 0-6 | NR |  | University hospital | Spain | Spanish | NR |
|  | 11 | 1608 | 72 |  |  | NR |  | Diabetes  (89% DM2) and comorbidity | 9 |  |  | NR |  | Diabetes clinic | USA | English | 53 |
|  | 12 | 1001 |  | NR |  | 49 |  | DM2 |  | NR |  | NR |  | Hospital | UK | English | NR |
| Sleep and Vitality Questionnaire (SVQ) | 13 | 73 | 58 | 5 |  | 63 |  | DM2 |  | NR |  | Insulin resistant |  | Hospital | UK | English | 83 |
|  | 14 | 512 |  |  | 40-85 | 46 |  | Diabetes  (93% DM2) | 8 | 2 | 2-14 | NR |  | Community health center | Australia | English | NR |
| Sleep Impact in Diabetes (SID) | 17 | 349 | 63 | 4 | 50-75 | NR |  | DM2 |  | NR |  | NR |  | Diabetes clinic | USA | English | 42 |
|  | *Responsiveness:* | *120* |  | *NR* |  | *NR* |  | *DM2* |  | *NR* |  | *NR* |  | *Diabetes clinic* | *USA* | *English* | *NR* |

Abbreviations: DM2: diabetes mellitus type 2; NR: not reported; SD: standard deviation
*Notes: If a subsample of the population is used for the assessment of some measurement properties, indicate this by adding sub rows (see example first and last row) or adding a footnote*

# **Template 6**

**Table.** Results on the risk of bias (RoB) and ratings for PROM development, content validity and reviewer ratings; and the summarized ratings and certainty of evidence for each PROM*

| **PROM** | **PROM development** | | | | | | | | | |  | **Content validity** | | | | | | | | | | | | | | **Reviewer ratings** | | | **Summarized ratings** | | | | | | | | **Comments^a^** |
| --- | --- | --- | --- | --- | --- | --- | --- | --- | --- | --- | --- | --- | --- | --- | --- | --- | --- | --- | --- | --- | --- | --- | --- | --- | --- | --- | --- | --- | --- | --- | --- | --- | --- | --- | --- | --- | --- |
|  |  | |  | |  |  | |  | |  |  | Patients | | | | | | | Professionals | | | | | | |  | | |  |  | | | | | | |  |
|  |  | |  | |  | Relevant | | | | |  | Relevant | | |  | | | |  | Relevant | | | | | | Relevant | |  | **Relevant** | | | |  | | |  |  |
|  | Concept elicitation | | Pilot  study | | Total  design | Comprehensive | | | | |  | Comprehensive | | | | | | |  | Comprehensive | | | | | | Comprehensive | | | **Comprehensive** | | | | | | | |  |
|  |  |  |  |  |  | Comprehensible | | | | |  |  | |  |  | Comprehensible | | |  |  |  |  | Comprehensible | | | Comprehensible | | |  |  |  | |  | **Comprehensible** | | |  |
|  | N | RoB | N | RoB | RoB | Rt | Rt | | Rt | | N | | RoB | Rt | RoB | Rt | RoB | Rt | N | RoB | Rt | RoB | Rt | RoB | Rt | Rt | Rt | Rt | **Rt** | **Certainty** | | **Rt** | **Certainty** | | **Rt** | **Certainty** |  |
| Amsterdam Quality of Life Questionnaire (AQOLQ) –  Fatigue subscale | 35 | D |  |  | D | +/– | + | | ? | |  | |  |  |  |  |  |  |  |  |  |  |  |  |  | + | + | + | **+** | **Moderate** | | **+** | **Moderate** | | **+** | **High** |  |
| Ref 4 |  |  |  |  |  |  |  | |  | | 32 | | V | + | V | + | V | + | 5 | A | + | A | + | A | ? |  |  |  |  |  |  |  |  |  |  |  |  |
| Ref 5 |  |  |  |  |  |  |  | |  | | 12 | |  |  |  |  | D | + |  |  |  |  |  |  |  |  |  |  |  |  |  |  |  |  |  |  |  |
| Ref 6 |  |  |  |  |  |  |  | |  | |  | |  |  |  |  |  |  | 15 |  |  | D | – |  |  |  |  |  |  |  |  |  |  |  |  |  |  |
| Diabetic Fatigue and Energy Scale (DFES) | 17 | I | 13 | D | I | +/– | + | | + | |  | |  |  |  |  |  |  |  |  |  |  |  |  |  | + | + | + | **+/**– | **Very low** | | **+** | **Very low** | | **+** | **Low** | Patients not involved in concept elicitation, no justification for recall period and response options |
| Sleep and Vitality Questionnaire (SVQ) |  |  | 8 | A | A | ? | ? | | ? | |  | |  |  |  |  |  |  |  |  |  |  |  |  |  | + | – | + | **+** | **Very low** | | **–** | **Very low** | | **+** | **Very low** | Key concepts missing |
| Sleep Impact in Diabetes (SID) | 8 | I |  |  | I | – | – | | ? | |  | |  |  |  |  |  |  |  |  |  |  |  |  |  | + | – | +/– | **+** | **Low** | | **–** | **Moderate** | | **+/**– | **Moderate** | Patients not involved in concept elicitation, key concepts missing, response options do not match items |
| Ref 16 |  |  |  |  |  |  |  | |  | | 24 | |  |  | A | – | A | + | 6 | D | + | D | – |  |  |  |  |  |  |  |  |  |  |  |  |  |  |

* Ratings: + sufficient; – insufficient; ± inconsistent; ? indeterminate; RoB: A adequate; D doubtful; I inadequate; V very good

^a^ If one of the summarized ratings is not sufficient: provide an explanation

Abbreviations: N: number; RoB: risk of bias; Rt: rating;

Blank space: study not conducted

# **Template 7**

**Note: this template is intentionally split ‘in the middle’ over 2 pages**

**Table.** Results on the risk of bias (RoB), raw results, and ratings for each study on a measurement property; and the summarized result, overall rating and level of evidence for each PROM

| **Amsterdam Quality of Life Questionnaire (AQOLQ) – Fatigue subscale** | | | | | | | | | | | | | |
| --- | --- | --- | --- | --- | --- | --- | --- | --- | --- | --- | --- | --- | --- |
| **Ref #** | **Country (language)** | **Structural validity** | | | **Internal consistency** | | | **Cross-cultural validity (CV)/  measurement invariance (MI)^a^** | | | **Reliability** | | |
|  |  | **N** | **RoB** | **Rating and *result*** | **N** | **RoB** | **Rating and *result*** | **N** | **RoB** | **Rating and *result*** | **N** | **RoB** | **Rating and *result*** |
| 7 | USA  (English) | 3635 | Very good | CTT +; IRT ?  Eight factor model:  *CFI 0.90*  *TLI 0.96*  *RMSEA 0.17*  *Resid corr <0.2*  *non-monotonic items deleted, model fit not reported* | 3635 | Very good | +  *Cronbach α 0.88*  *IRT reliab 0.90* | MI 3635 | MI Doubtful | MI: + *No DIF in any items for age, sex, ethnicity, race* | 100 | Adequate | +  *ICC 0.73* |
| 8 | USA  (English) | 1278 | Very good | CTT: +; IRT: –  Eight factor model:  *CFI 0.97*  *TLI 0.97*  *RMSEA 0.05*  *SRMR 0.07*  *Resid corr <0.2*  *Scalability >0.3 3 items with misfit (p<0.0001)* | 1278 | Very good | +  *Cronbach α 0.87*  *IRT reliab 0.9* | MI 1278  CV 1278 | MI Doubtful  CV Doubtful | MI: +; CV: –  *No DIF in any items for age or sex;  1 item DIF  for language* |  |  | NA |
| 9 | Netherlands (Dutch) |  |  | NA | 182 | Very good | –  *Cronbach α 0.66* |  |  | NA |  |  | NA |
| **Total sample size, certainty of evidence, overall rating, pooled or summarized result** | | **4913** | **Moderate:**  2 very good studies, serious inconsistency | **+**  *Unidimensional scale  (6 items)* | **5059** | **Moderate:** 3 very good studies | **+** *Cronbach α 0.66-0.88, majority >0.7* | **MI 4913**  **CV 1278** | **MI Moderate:**  2 doubtful studies  **CV Low:**  1 doubtful study | **MI: +**  *No DIF in any items for age, sex, ethnicity, race*  **CV: –**  *DIF for language in one item* | **100** | **Moderate:**  1 adequate study | **+**  *ICC 0.73* |

+ sufficient; – insufficient; ± inconsistent; ? indeterminate

Abbreviations: CFI: comparative fit index; CTT: classical test theory; CV: cross-cultural validity; DIF: differential item functioning; ICC: intraclass correlation coefficients; IRT: item response theory; MI: measurement invariance; N: number; NA not assessed; Resid corr: residual correlation; RMSEA: root mean square error of approximation; RoB: risk of bias; SRMR: standardized root mean square residual; TLI: Tucker-Lewis index

| **Amsterdam Quality of Life Questionnaire (AQOLQ) – Fatigue subscale** | | | | | | | | | | | | | |
| --- | --- | --- | --- | --- | --- | --- | --- | --- | --- | --- | --- | --- | --- |
| **Ref #** | **Country (language)** | **Measurement error** | | | **Criterion Validity** | | | **Hypothesis testing^:^ comparisons between instruments (CI) and known groups (KG)** | | | **Responsiveness** | | |
|  |  | **N** | **RoB** | **Rating and *result*** | **N** | **RoB** | **Rating and *result*** | **N** | **RoB** | **Rating and *result*** | **N** | **RoB** | **Rating and *result*** |
| 7 | USA  (English) | 3635 | Inadequate | –  *SEM 6.3;  SDC 17.5;  MIC 13.5* | 3635 | Very good | +  *r 0.95* |  |  | NA |  |  | NA |
| 8 | USA  (English) |  |  | NA |  |  | NA | CI 500- 1278  KG 1278 | CI Very good  KG Very good | CI ±  *10–/12+*  KG +  *6+* |  |  | NA |
| 9 | Netherlands (Dutch) |  |  | NA |  |  | NA | KG  73-182 | KG Very good | KG ±  *2–/4+* | 74-182 | Very good | ±  *3–/5+* |
| **Total sample size, certainty of evidence, overall rating, pooled or summarized result** | | **3635** | **Very low:**  1 inadequate study | **–**  *SDC < MIC* | **3635** | **High:**  1 very good study | **+**  *r 0.95* | **CI 500-1278**  **KG 1351- 1460** | **CI High:** 1 very good study  **KG Moderate:**  2 very good studies, serious inconsistency | **CI ±**  *10–/12+*  **KG +**  *2–/10+* | **74-182** | **Moderate:**  1 very good study, small sample size | **±**  *3–/5+* |

+ sufficient; – insufficient; ± inconsistent; ? indeterminate

Abbreviations: CI: comparisons between instruments; KG: known groups; MIC: minimal important change; N: number; NA not assessed; r: correlation; RoB: risk of bias; SDC: smallest detectable change; SEM: standard error of measurement

# **Template 8**

**Table.** Summary of findings with the evidence for each measurement property for each PROM*

| **Measurement property** | **Amsterdam Quality of Life Questionnaire (AQOLQ) –**  **Fatigue subscale** | | **Diabetic Fatigue and Energy Scale (DFES)** | | **Sleep and Vitality Questionnaire (SVQ)** | | **Sleep Impact in Diabetes (SID)** | |
| --- | --- | --- | --- | --- | --- | --- | --- | --- |
|  | **Overall rating** | **Certainty of evidence** | **Overall rating** | **Certainty of evidence** | **Overall rating** | **Certainty of evidence** | **Overall rating** | **Certainty of evidence** |
| Content validity |  |  |  |  |  |  |  |  |
| *Relevance* | + | Moderate | +/– | Very low | + | Very low | + | Low |
| *Comprehensiveness* | + | Moderate | + | Very low | – | Very low | – | Moderate |
| *Comprehensibility* | + | High | + | Low | + | Very low | +/– | Moderate |
| Structural validity | + | Moderate | + | High | + | Low | + | High |
| Internal consistency | + | Moderate | + | High | + | Low | + | High |
| Cross-cultural validity | + | Moderate |  |  |  |  |  |  |
| Measurement invariance | + | Moderate | + | Low | + | Moderate | – | Low |
| Reliability | + | Moderate | + | Moderate |  |  |  |  |
| Measurement error | – | Very low |  |  |  |  | ? |  |
| Criterion validity | + | High |  |  | + | High |  |  |
| Construct: known groups | +/– | High | + | Moderate | +/– | High |  |  |
| Construct: other instruments | + | Moderate | +/– | High | +/– | Low | ? |  |
| Responsiveness | +/– | Moderate | – | High |  |  | +/– | Very low |

* Colors represent sufficiency of measurement properties, shading represents quality of the evidence:

Green: sufficient; red: insufficient yellow: inconsistent; grey: indeterminate; darker shading: higher quality evidence

Blank space: lack of evidence

# **Template 8a – alternative**

**Table.** Summary of findings with the evidence for each measurement property for each PROM*

| **Measurement property** | | |  |  |  | Structural validity | | Cross-cultural validity | | Reliability | | Criterion validity | | Responsiveness | |
| --- | --- | --- | --- | --- | --- | --- | --- | --- | --- | --- | --- | --- | --- | --- | --- |
|  |  | Content validity | | | |  | Internal  consistency | | Measurement invariance | | Measurement  error | | Construct validity | |  |
|  |  | *Overall* | *Comprehensiveness* | | |  |  |  |  |  |  |  | *Known* | *Other* |  |
| Name PROM | **Ratings** |  | *Relevance* | *Comprehensibility* | |  |  |  |  |  |  |  | *groups* | *instruments* | |
| **Amsterdam Quality of Life Questionnaire (AQOLQ) –**  **Fatigue subscale** | **Overall** |  | + | + | + | + | + | + | + | + | – | + | +/– | + | +/– |
|  | **Certainty** |  | Moderate | Moderate | High | Moderate | Moderate | Moderate | Moderate | Moderate | Very low | High | High | Moderate | Moderate |
| **Diabetic Fatigue and Energy Scale (DFES)** | **Overall** |  | +/– | + | + | + | + |  | + | + |  |  | + | +/– | – |
|  | **Certainty** |  | Moderate | Very low | Low | High | High |  | Low | Moderate |  |  | Moderate | High | High |
| **Sleep and Vitality Questionnaire (SVQ)** | **Overall** |  | + | – | + | + | + |  | + |  |  | + | +/– | +/– |  |
|  | **Certainty** |  | Very low | Very low | Very low | Low | Low |  | Moderate |  |  | High | High | Low |  |
| **Sleep Impact in Diabetes (SID)** | **Overall** |  | + | – | +/– | + | + |  | – |  | ? |  |  | ? | +/– |
|  | **Certainty** |  | Very low | Moderate | Moderate | High | High |  | Low |  |  |  |  |  | Very low |

* Colors represent sufficiency of measurement properties, shading represents quality of the evidence:

Green: sufficient; red: insufficient yellow: inconsistent; grey: indeterminate; darker shading: higher quality evidence

Blank space: lack of evidence

1. Elsman, E.B.M, et al., *Guideline for reporting systematic reviews of outcome measurement instruments (OMIs): PRISMA-COSMIN for OMIs 2024*. Journal of Clinical Epidemiology, 2024. [↑](#footnote-ref-1)
2. Mokkink L.B., Elsman, E.B.M., and Terwee, C.B., *COSMIN guideline for systematic reviews of patient-reported outcome measures version 2.0.* Qual Life Res, 2024. **33**(11): p. 2929-2939. [↑](#footnote-ref-2)
